# Supplementary material for: Plug-and-play evolution of the Klebsiella pneumoniae capsule locus enables serotype exchange across genetic backgrounds
Source: PLoS Biol. 2026 Mar 25;24(3):e3003724. doi: 10.1371/journal.pbio.3003724 (PMC13043062; doi:10.1371/journal.pbio.3003724)
Supplement: S1 Fig — Capsule locus identification and annotation were done using Kaptive [36]. Alignment and visualization were done using Clinker [92] (https://github.com/gamcil/clinker) with modifications. Small gray arrows indicate promoters of the locus. Large gray arrows represent nonannotated proteins with no homologs in the other capsule loci, whereas arrows in shades of dark blue correspond to hypothetical proteins with homologs in other strains. K types labeled as ‘Alternative loci’, corresponding to Kpn NTUH K2044 and Kpn CIP 52.145, are native loci to their strains but not used as template to generate the capsule-swapped strains. (DOCX) [file pbio.3003724.s001.docx]

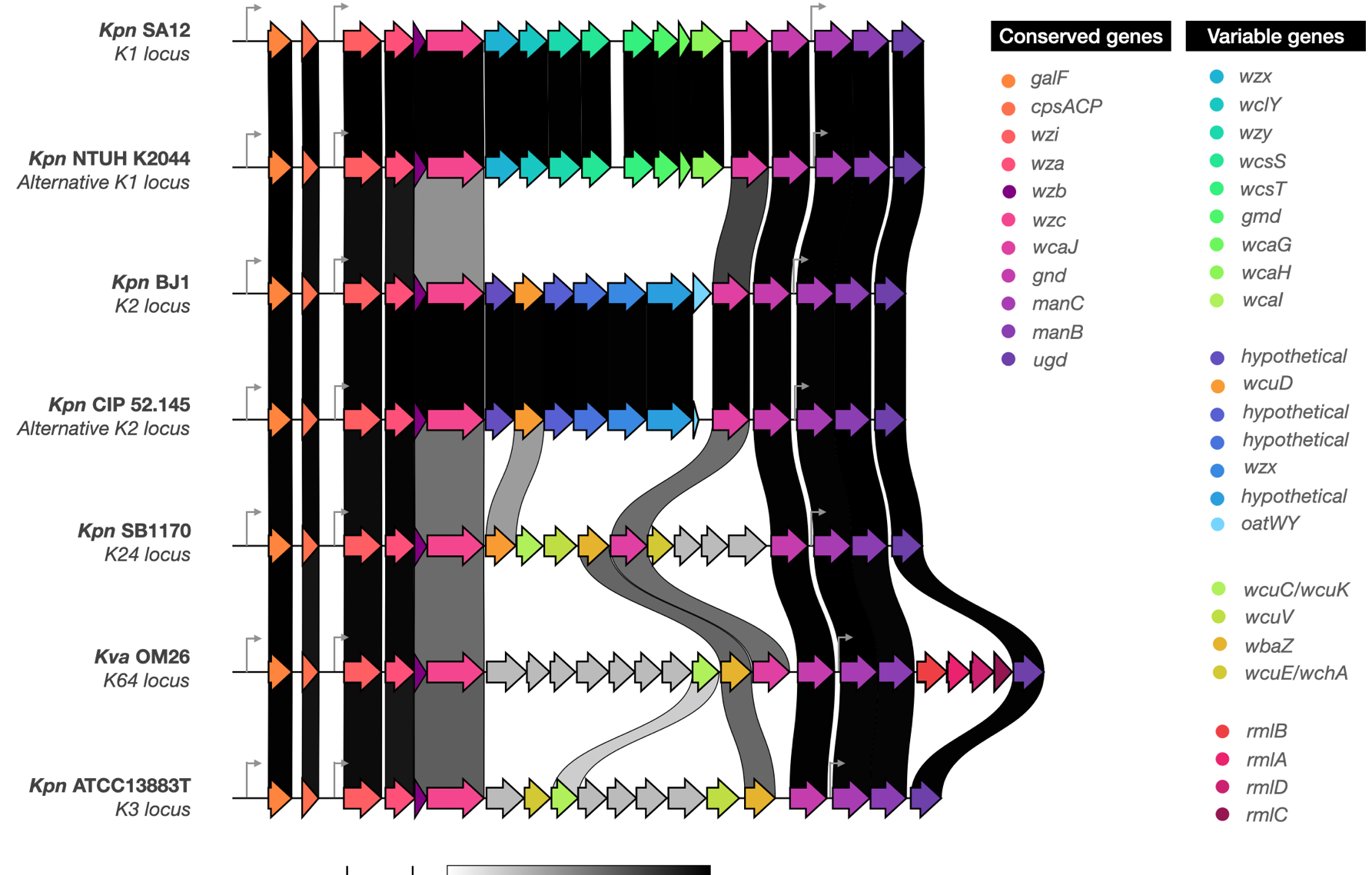

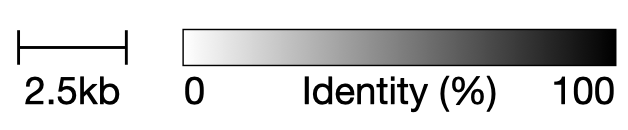


**S1 Fig. Genomic organization of capsule loci of strains used in the study.** Capsule locus identification and annotation were done using Kaptive^1^. Alignment and visualization were done using Clinker^2^ (<https://github.com/gamcil/clinker>) with modifications. Small grey arrows indicate promoters of the locus. Large grey arrows represent non-annotated proteins with no homologs in the other capsule loci, whereas arrows in shades of dark blue correspond to hypothetical proteins with homologs in other strains. K types labelled as ‘Alternative loci’, corresponding to *Kpn* NTUH K2044 and *Kpn* CIP 52.145, are native loci to their strains but not used as template to generate the capsule-swapped strains.

### REFERENCES

1. Stanton, T. D., Hetland, M. A. K., Löhr, I. H., Holt, K. E. & Wyres, K. L. Fast and accurate in silico antigen typing with Kaptive 3. *Microb. Genomics* **11**, (2025).

2. Gilchrist, C. L. M. & Chooi, Y.-H. clinker & clustermap.js: automatic generation of gene cluster comparison figures. *Bioinformatics* **37**, 2473–2475 (2021).
