# Supplementary material for: Plug-and-play evolution of the Klebsiella pneumoniae capsule locus enables serotype exchange across genetic backgrounds
Source: PLoS Biol. 2026 Mar 25;24(3):e3003724. doi: 10.1371/journal.pbio.3003724 (PMC13043062; doi:10.1371/journal.pbio.3003724)
Supplement: S2 Fig — A. Comparison of capsule production between the wild type strain and its complemented strain corresponding to the reintroduction of either the native capsule locus (green) or an alternative capsule locus of the same K type (pink). Values were normalized by an OD600 of 1. P-values correspond to unpaired Wilcoxon test. B and C. Capsule production across different capsule loci (B) or genetic background (C), ranked from lowest to highest capsule production. Blue lines represent regressions for each K type (R2), and the surrounding gray area indicate the standard error. Each dot corresponds to an independent biological replicate. The data underlying this Figure can be found in S2 Data. (DOCX) [file pbio.3003724.s002.docx]

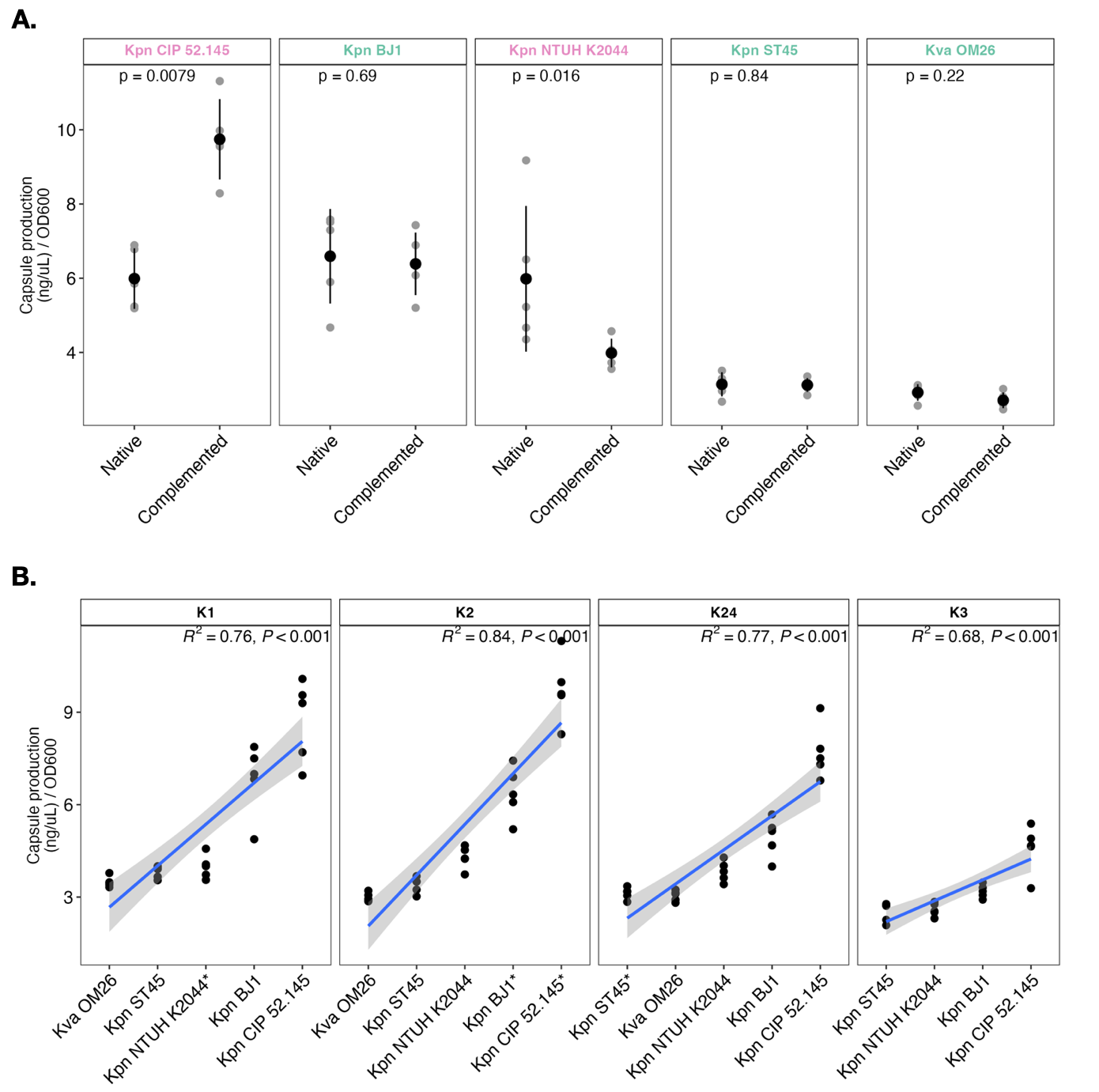

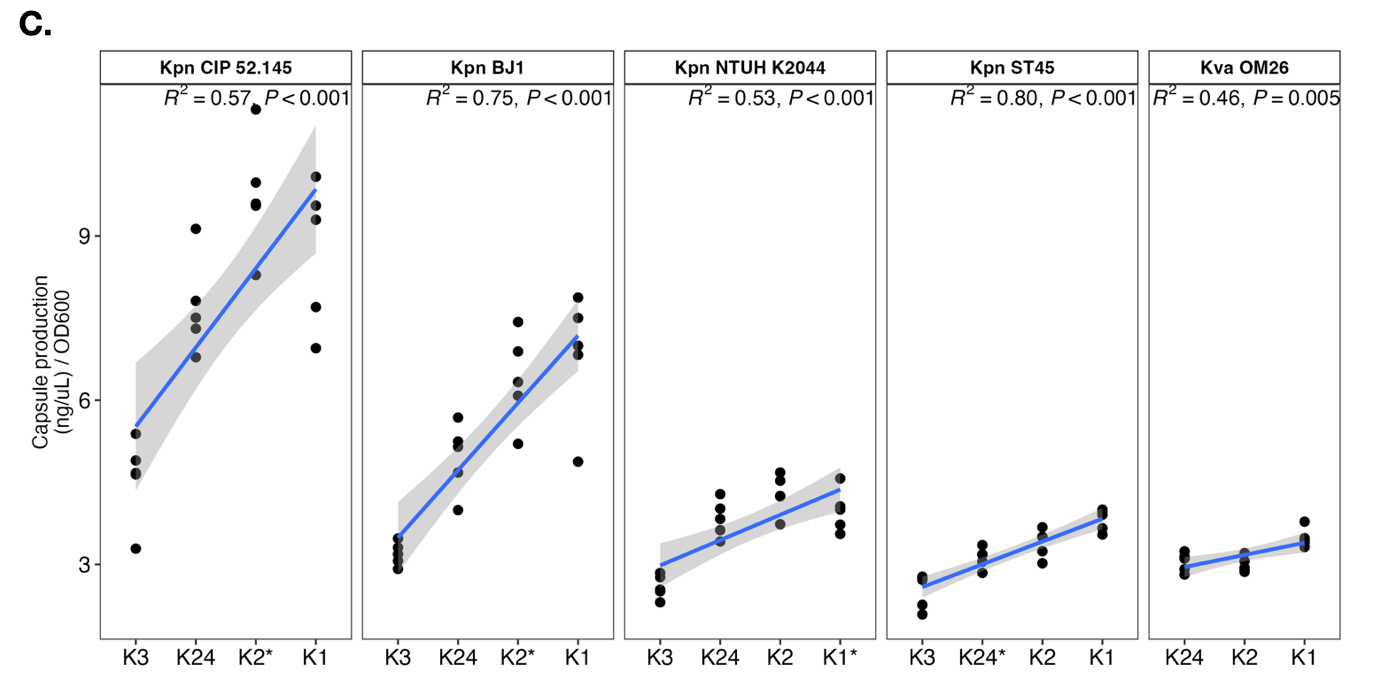


**S2 Fig. Capsule production of capsule-swapped strains determined by the uronic acid method.** **A.** Comparison of capsule production between the wild type strain and its complemented strain corresponding to the reintroduction of either the native capsule locus (green) or an alternative capsule locus of the same K type (pink). Values were normalized by an OD_600_ of 1. P-values correspond to unpaired Wilcoxon test. **B and C.** Capsule production across different capsule loci (**B**) or genetic background (**C**), ranked from lowest to highest capsule production. Blue lines represent regressions for each K type (R^2^), and the surrounding grey area indicate the standard error. Each dot corresponds to an independent biological replicate. The data underlying this Figure can be found in S2 Data.
