# Supplementary material for: Plug-and-play evolution of the Klebsiella pneumoniae capsule locus enables serotype exchange across genetic backgrounds
Source: PLoS Biol. 2026 Mar 25;24(3):e3003724. doi: 10.1371/journal.pbio.3003724 (PMC13043062; doi:10.1371/journal.pbio.3003724)
Supplement: S3 Fig — A. Representative images of Klebsiella strains under the microscope. India ink staining images of Kpn CIP 52.145, Kpn NTUH K2044, and Kpn ST45 at a magnification of 100× after overnight culture in LB. Scale bar = 10 μm. B. Capsule area positively correlates with capsule production. Each panel represents each set of capsule-swapped from the three independent genetic backgrounds measured. Capsule thickness was determined by measuring the exclusion area generated by the capsule. This area was quantified from microscopic images of India ink-stained cells using ImageJ. The lightly capsulated cells could not be measured confidently and are thus not included in the capsule thickness analyses. The size of points indicates the number of cells analyzed (between 50 and 100, except for the very lightly capsulated strains). Capsule production was estimated by glucuronic acid method from capsule extracts (see Materials and methods). C. Correlation of all capsule-swapped strains together, irrespective of their genetic background. Statistical analyses for panels B and C were performed using a linear model (LM) implemented with the smooth function in R. The data underlying this Figure can be found in S2 Data. (DOCX) [file pbio.3003724.s003.docx]

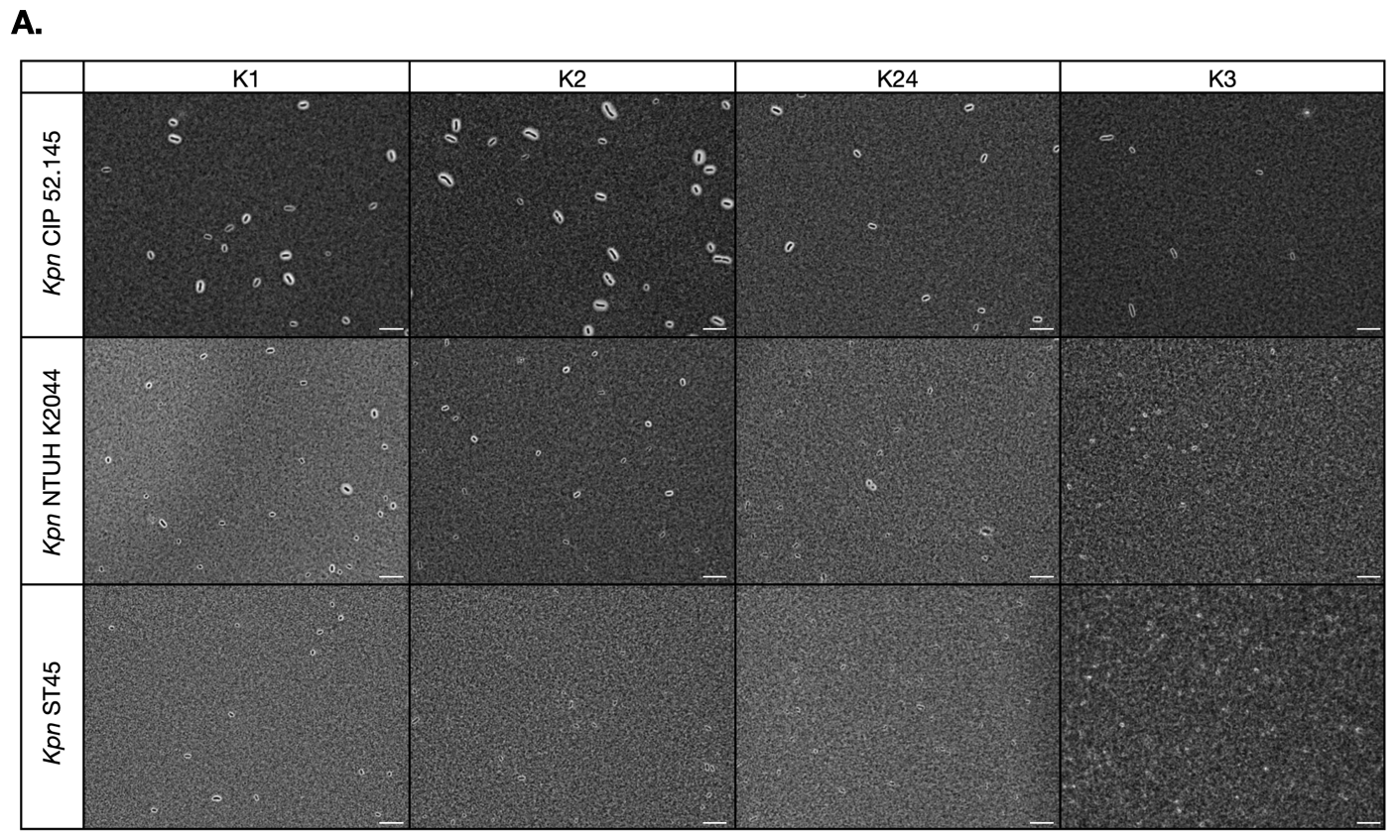

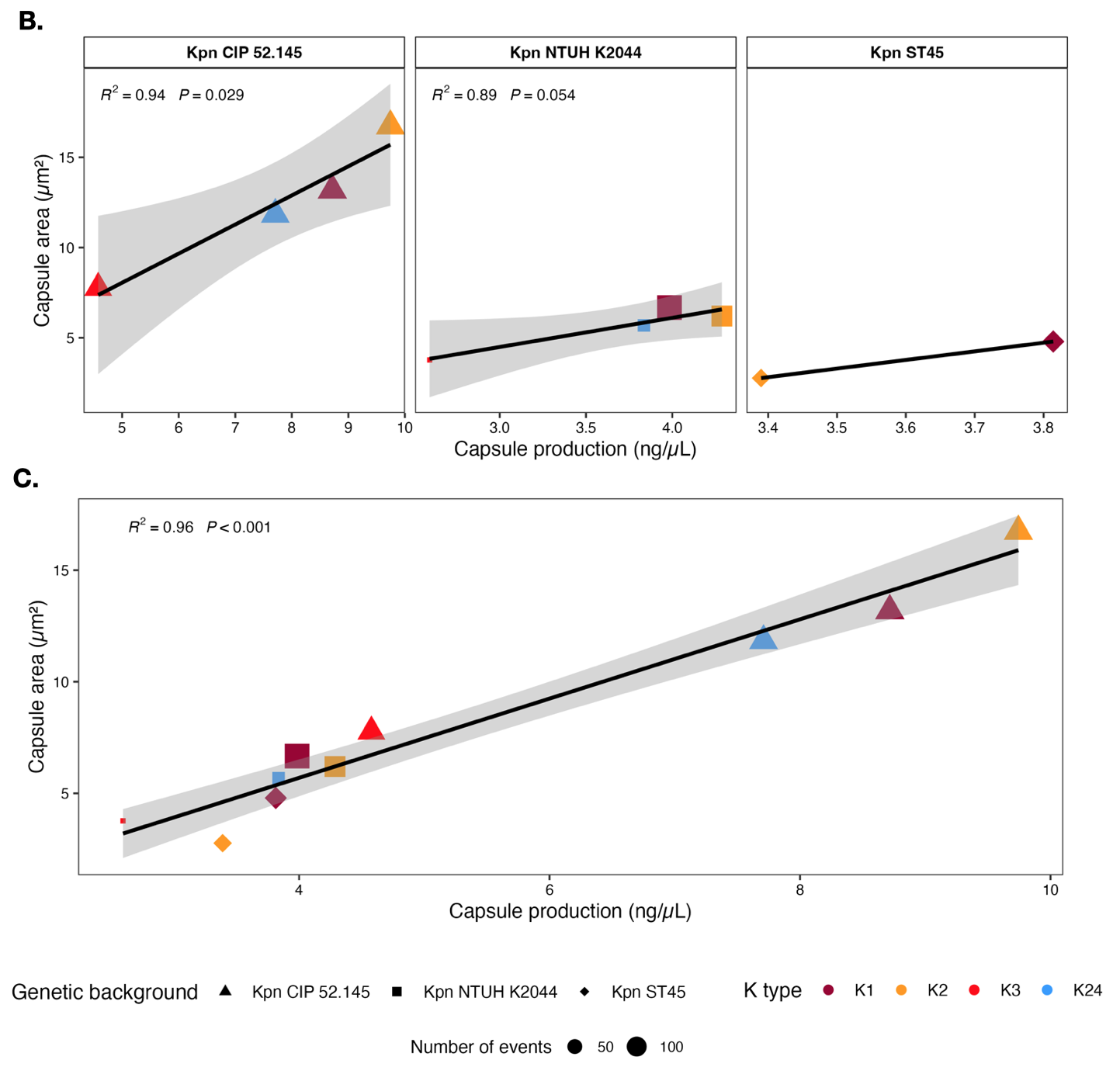


**S3 Fig. Correlation between capsule thickness and capsule quantification. A**. Representative images of *Klebsiella* strains under the microscope. India ink staining images of Kpn CIP 52.145, Kpn NTUH K2044 and Kpn ST45 at a magnification of 100× after overnight culture in LB. Scale bar = 10 μm. **B.** Capsule area positively correlates with capsule production. Each panel represents each set of capsule-swapped from the three independent genetic backgrounds measured. Capsule thickness was determined by measuring the exclusion area generated by the capsule. This area was quantified from microscopic images of India ink-stained cells using ImageJ. The lightly capsulated cells could not be measured confidently and are thus not included in the capsule thickness analyses. The size of points indicates the number of cells analyzed (between 50 to 100, except for the very lightly capsulated strains). Capsule production was estimated by glucuronic acid method from capsule extracts (see methods). **C.** Correlation of all capsule-swapped strains together, irrespective of their genetic background. Statistical analyses for panels B and C were performed using a linear model (LM) implemented with the *smooth* function in R. The data underlying this Figure can be found in S2 Data.
