# Supplementary material for: Plug-and-play evolution of the Klebsiella pneumoniae capsule locus enables serotype exchange across genetic backgrounds
Source: PLoS Biol. 2026 Mar 25;24(3):e3003724. doi: 10.1371/journal.pbio.3003724 (PMC13043062; doi:10.1371/journal.pbio.3003724)
Supplement: S4 Fig — Log2FC (fold change) of capsule locus genes, i.e., genes present in any of the five capsule loci types considered. The shape indicates the significance of the adjusted p-value and the color represents the capsule types. The genetic background and the native capsule types are indicated at the top of each subpanel. Capsule genes indicated on the x-axis are colored considering their K type specificity: dark red for K1-specific genes, wzy is indicated in brown (identified for K1, K24, and K64), orange for K2, light blue for K24, red for K3, wbaZ is indicated in purple (identified in K3, K24, and K64 serotypes) and dark blue for K64-specific genes. Core capsule genes (present in all K types) are in black. The data underlying this Figure can be found in S2 Data. (DOCX) [file pbio.3003724.s004.docx]

**
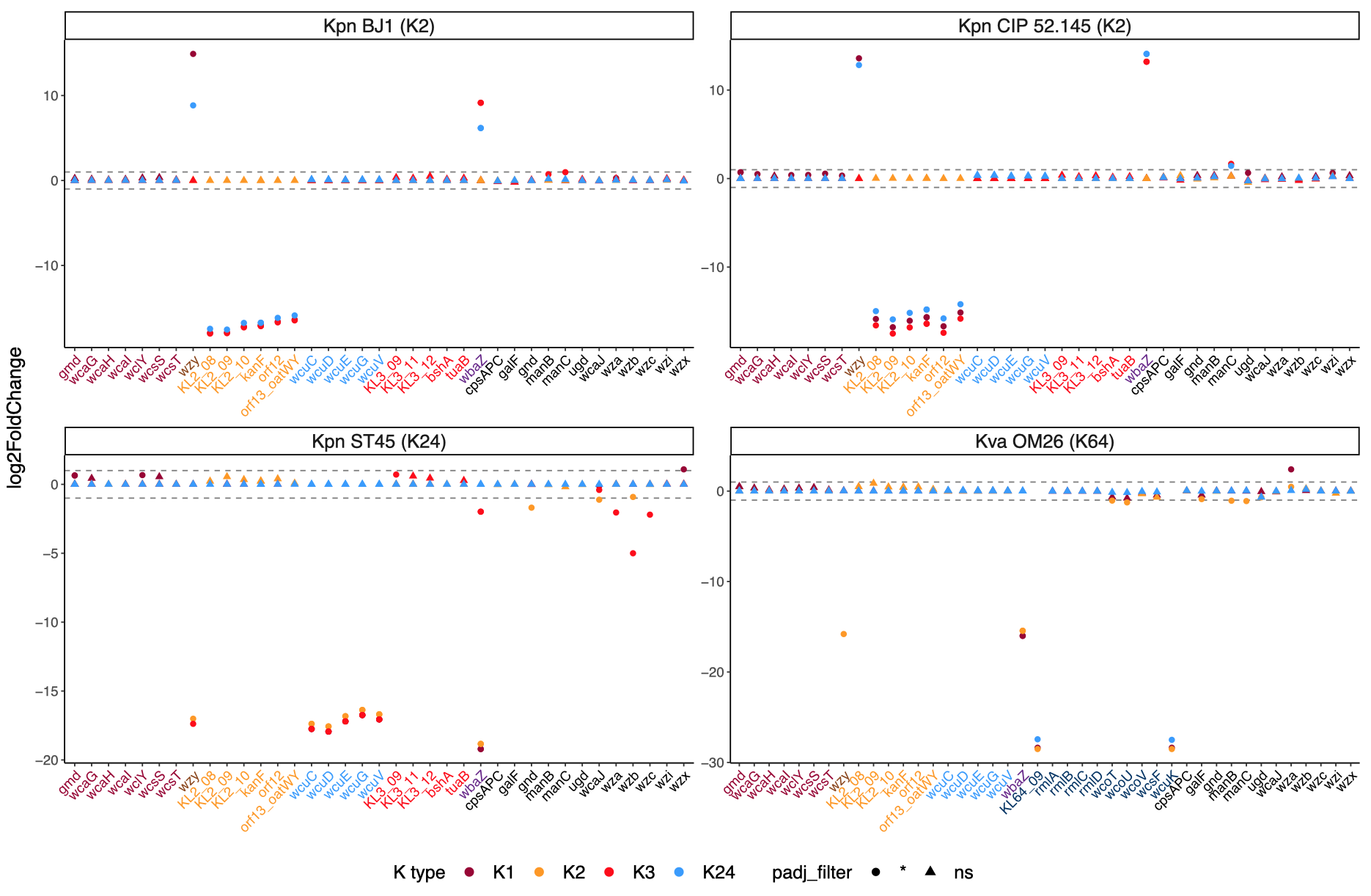
**

**S4 Fig. Transcriptomic analyses of capsule loci genes in capsule-swapped strains.** Log_2_FC (fold change) of capsule locus genes, i.e. genes present in any of the five capsule loci types considered. The shape indicates the significance of the adjusted p-value and the color represents the capsule types. The genetic background and the native capsule types are indicated at the top of each subpanel. Capsule genes indicated on the x-axis are colored considering their K type specificity: dark red for K1-specific genes, *wzy* is indicated in brown (identified for K1, K24 and K64), orange for K2, light blue for K24, red for K3, *wbaZ* is indicated in purple (identified in K3, K24 and K64 serotypes) and dark blue for K64-specific genes. Core capsule genes (present in all K types) are in black. The data underlying this Figure can be found in S2 Data.
