# Supplementary material for: Plug-and-play evolution of the Klebsiella pneumoniae capsule locus enables serotype exchange across genetic backgrounds
Source: PLoS Biol. 2026 Mar 25;24(3):e3003724. doi: 10.1371/journal.pbio.3003724 (PMC13043062; doi:10.1371/journal.pbio.3003724)
Supplement: S5 Fig — Components 1 and 2 are displayed and are followed by the percentage of variance explained by each component. Each dot represents a biological replicate annotated as follows: replicate number (EX), underscore and K type (KX). PCA analysis was performed on the core capsule genes (A and B) and the core genome (C). For better visualization of the clustering of capsule-swapped strains, the PCA on the core capsule was done with (A) and without (B) taking into account the dCap (acapsulated) strains. The data underlying this Figure can be found in S2 Data. (DOCX) [file pbio.3003724.s005.docx]

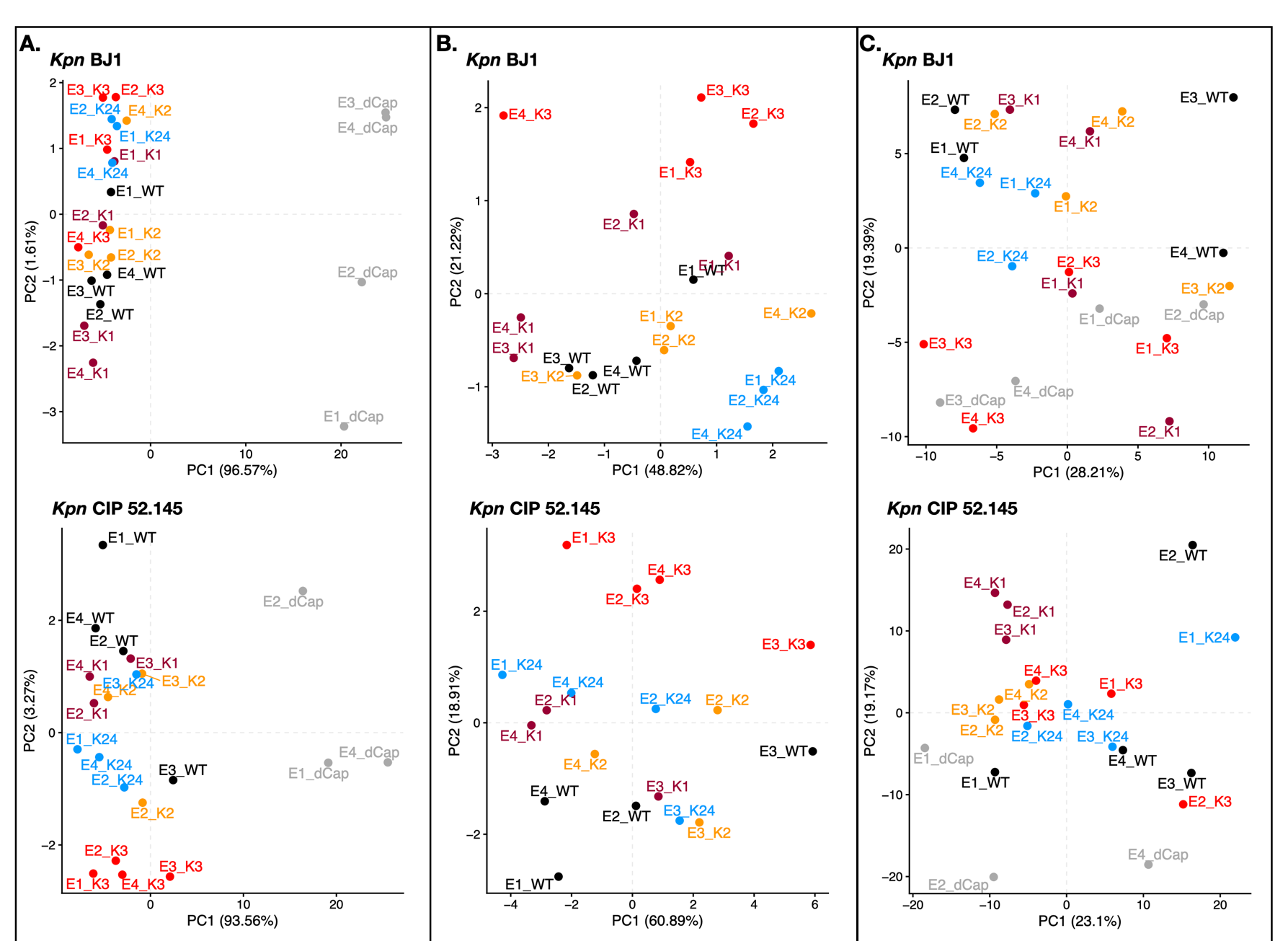

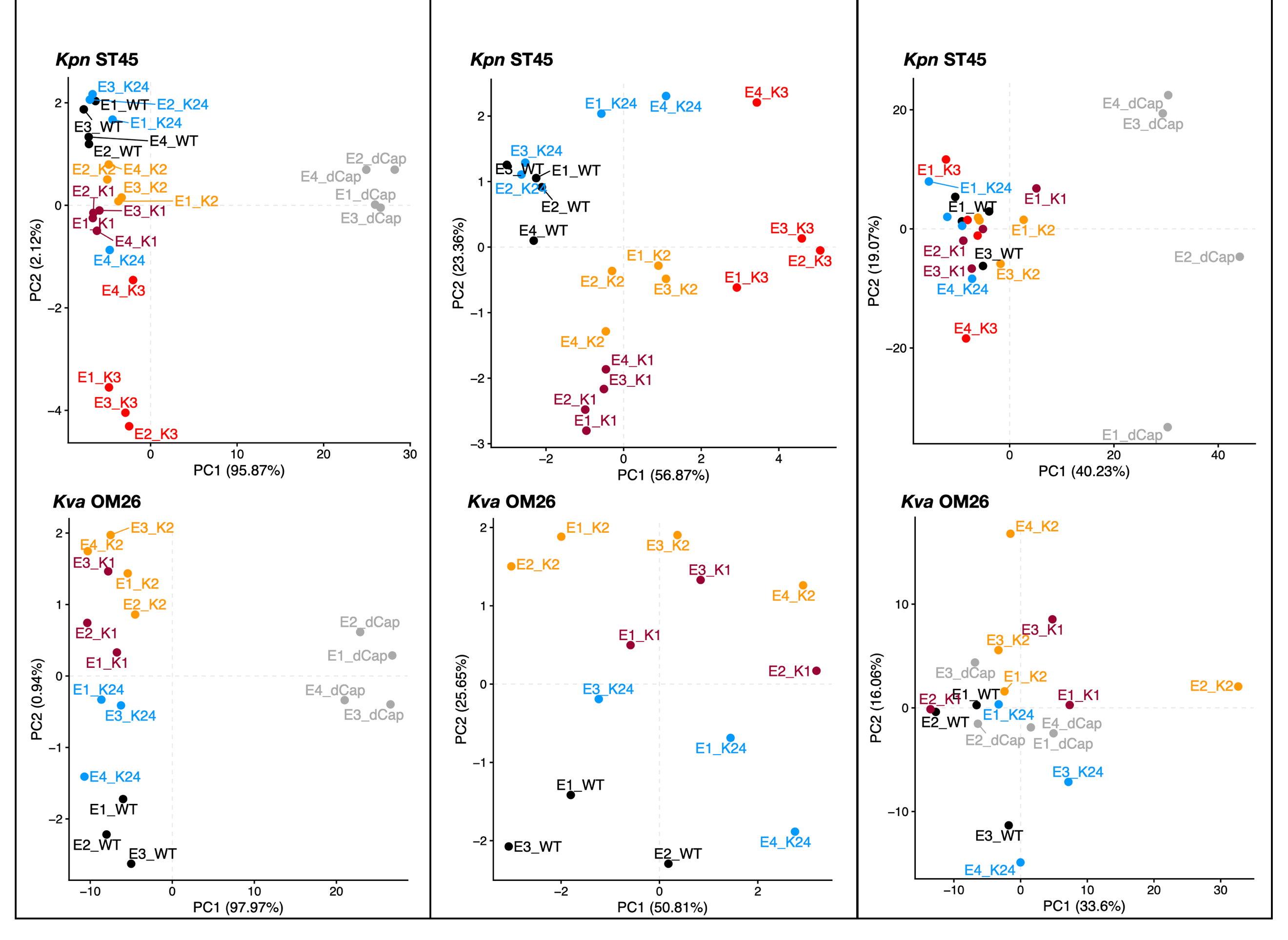


**S5 Fig. Adjusted Principal Component Analysis (PCA) of gene expression.** Components 1 and 2 are displayed and are followed by the percentage of variance explained by each component. Each dot represents a biological replicate annotated as follows: replicate number (EX), underscore and K type (KX). PCA analysis was performed on the core capsule genes (**A-B**) and the core genome (**C**). For better visualization of the clustering of capsule-swapped strains, the PCA on the core capsule was done with (**A**) and without (**B**) taking into account the dCap (acapsulated) strains. The data underlying this Figure can be found in S2 Data.
