# Supplementary material for: Plug-and-play evolution of the Klebsiella pneumoniae capsule locus enables serotype exchange across genetic backgrounds
Source: PLoS Biol. 2026 Mar 25;24(3):e3003724. doi: 10.1371/journal.pbio.3003724 (PMC13043062; doi:10.1371/journal.pbio.3003724)
Supplement: S6 Fig — A and B. Growth curves (error bars are not included for visibility reasons) (A) and the area under the growth curve (AUC) (B) of capsule-swapped strains relative to their respective native strain (dotted line) in either nutrient-rich (LB) or nutrient-poor (M02) media. Gray dots represent individual biological replicates (N = 5). C. Pairwise competitions in nutrient-poor medium (M02) of fluorescent versus nonfluorescent strain (one-sample t test, difference from 1). Asterisk beside K loci (*) indicates the native serotype of each strain. ∆cap indicate acapsulated control strains. The data underlying this Figure can be found in S2 Data. (DOCX) [file pbio.3003724.s006.docx]

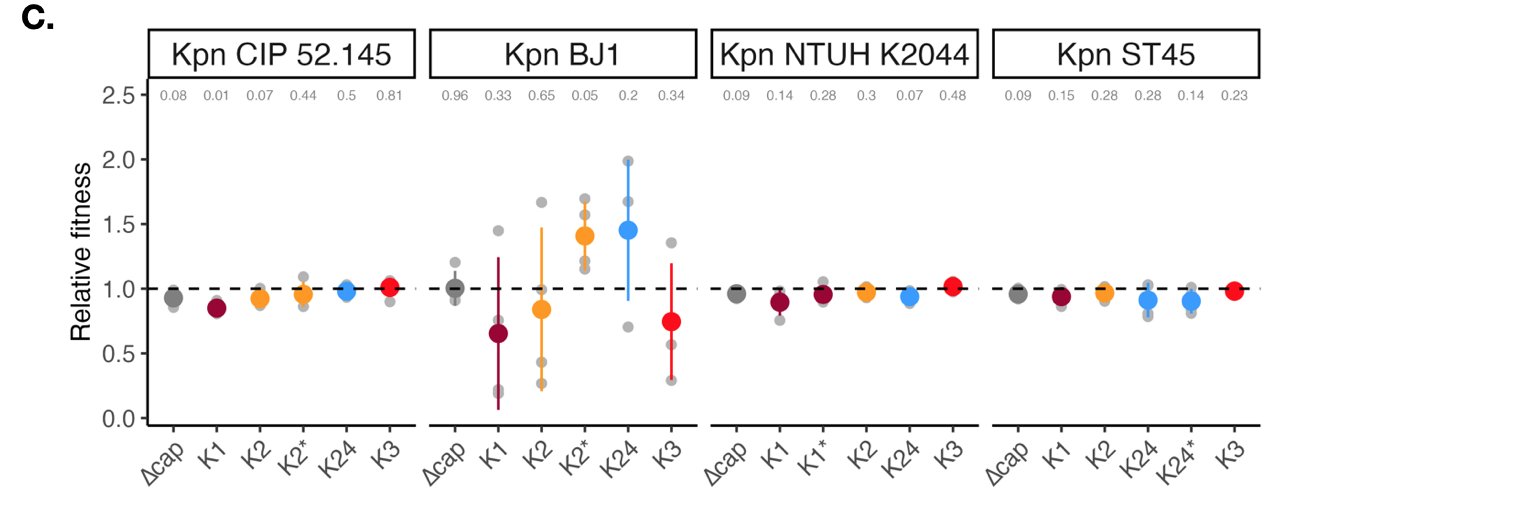

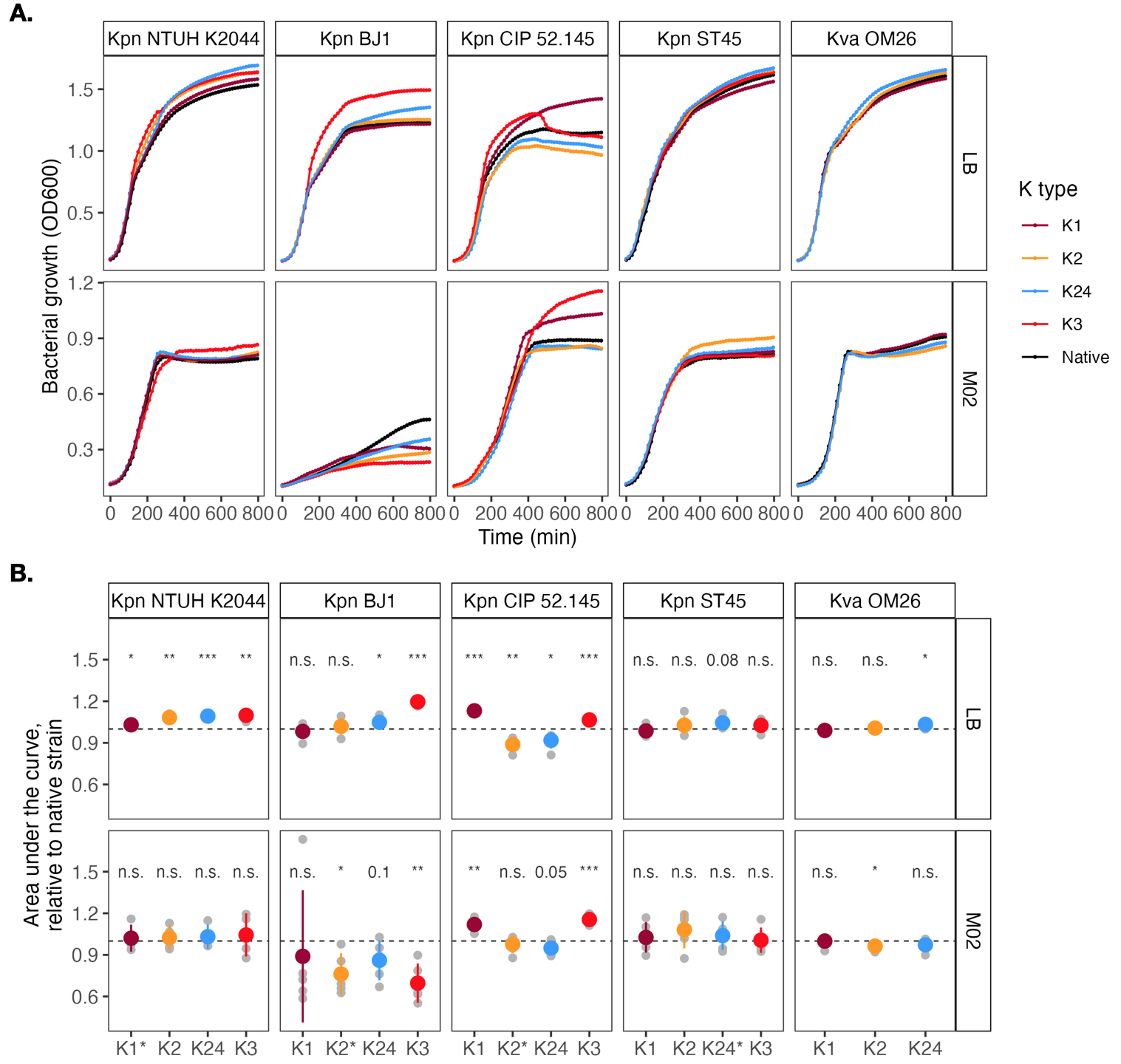


**S6 Fig. Growth and fitness of capsule-swapped strains. A-B.** Growth curve raw data (error bars are not included for visibility reasons) (**A**) and the areas under the growth curve (AUC) (**B**) of capsule-swapped strains relative to their respective native strain (dotted line) in either nutrient-rich (LB) or nutrient-poor (M02) media. Grey dots represent individual biological replicates (N=5). **C.** Pairwise competitions in nutrient-poor medium (M02) of fluorescent versus non-fluorescent strain (one-sample t-test, difference from 1). Asterisk beside K loci (*) indicates the native serotype of each strain. ∆cap indicate acapsulated control strains. The data underlying this Figure can be found in S2 Data.
