# Supplementary material for: Plug-and-play evolution of the Klebsiella pneumoniae capsule locus enables serotype exchange across genetic backgrounds
Source: PLoS Biol. 2026 Mar 25;24(3):e3003724. doi: 10.1371/journal.pbio.3003724 (PMC13043062; doi:10.1371/journal.pbio.3003724)
Supplement: S7 Fig — A. Proportion of capsulated clones throughout the 15 days of evolution of parental strains and their respective isogenic capsule-swapped strains before daily passages of each culture either in nutrient-rich (LB, green line) or nutrient-poor (M02, blue line) media. Bold lines represent the average of the independent populations of the same strain grown in a given environment. Gray lines represent each of the independent populations. B. Proportion of capsulated clones throughout the first 5 days of evolution in nutrient-rich medium. Each line represents the average of at least three independently-evolving populations. The data underlying this Figure can be found in S2 Data. (DOCX) [file pbio.3003724.s007.docx]

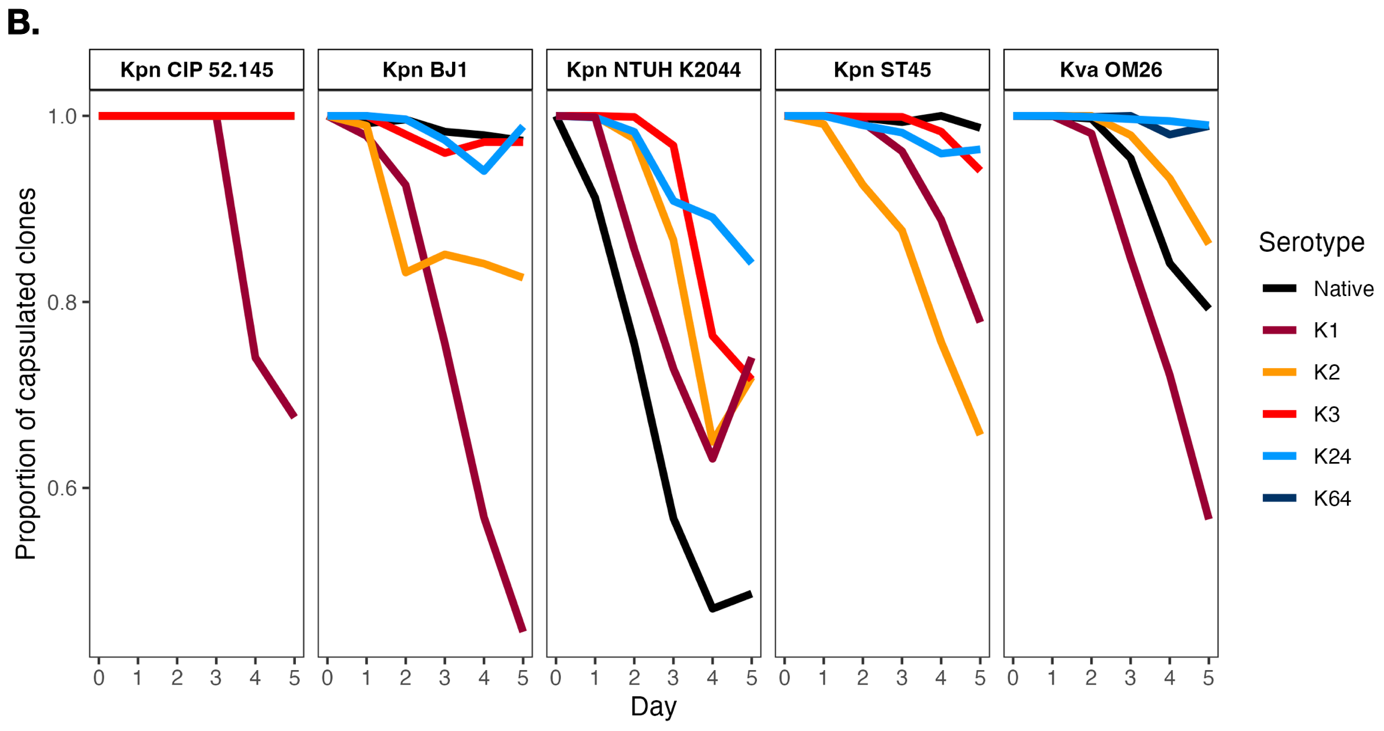

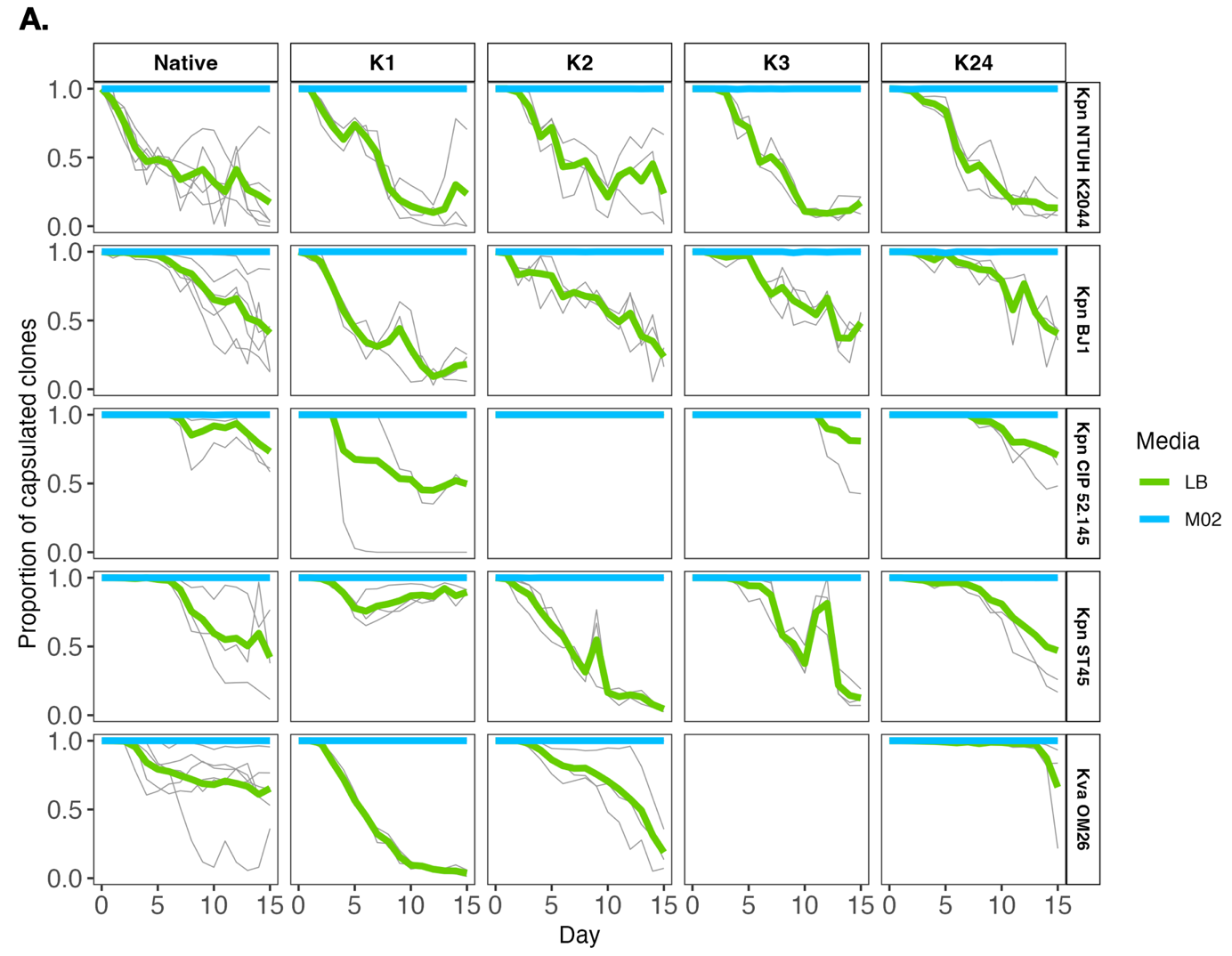


**S7 Fig. Evolutionary fate of the capsule in capsule-swapped and native strains.** **A.** Proportion of capsulated clones throughout the fifteen days of evolution of parental strains and their respective isogenic capsule-swapped strains before daily passages of each culture either in nutrient-rich (LB, green line) or nutrient-poor (M02, blue line) media. Bold lines represent the average of the independent populations of the same strain grown in a given environment. Grey lines represent each of the independent populations. **B.** Proportion of capsulated clones throughout the first five days of evolution in nutrient-rich medium. Each line represents the average of at least three independently evolving populations. The data underlying this Figure can be found in S2 Data.
