# Supplementary material for: Plug-and-play evolution of the Klebsiella pneumoniae capsule locus enables serotype exchange across genetic backgrounds
Source: PLoS Biol. 2026 Mar 25;24(3):e3003724. doi: 10.1371/journal.pbio.3003724 (PMC13043062; doi:10.1371/journal.pbio.3003724)
Supplement: S8 Fig — A and B. Capsule-swapped strain’s survival to 0.5% cholate (CHO) or deoxycholate (DCO) (A) and to 5 mM or 10 mM H2O2 (B), relative to their respective untreated condition. *p < 0.05; **p < 0.01; ***p < 0.001, one-sample t test, difference from 100. C. Native capsulated strains and their respective acapsular mutant (∆cap) survival to 5 or 10 mM H2O2, relative to their respective nontreated condition. ns, nonsignificant, two-sample paired t test. D. Biofilm formation of capsule-swapped strains in nutrient-rich (LB) or nutrient-poor (M02) media, relative to their respective native strain. Shape of dots correspond to the genetic background; the K type is indicated on the x-axis and identified by the color. Each point represents the mean of at least three independent biological replicates. ns: nonsignificant; one-sample t test, difference from 1. The data underlying this Figure can be found in S2 Data. (DOCX) [file pbio.3003724.s008.docx]

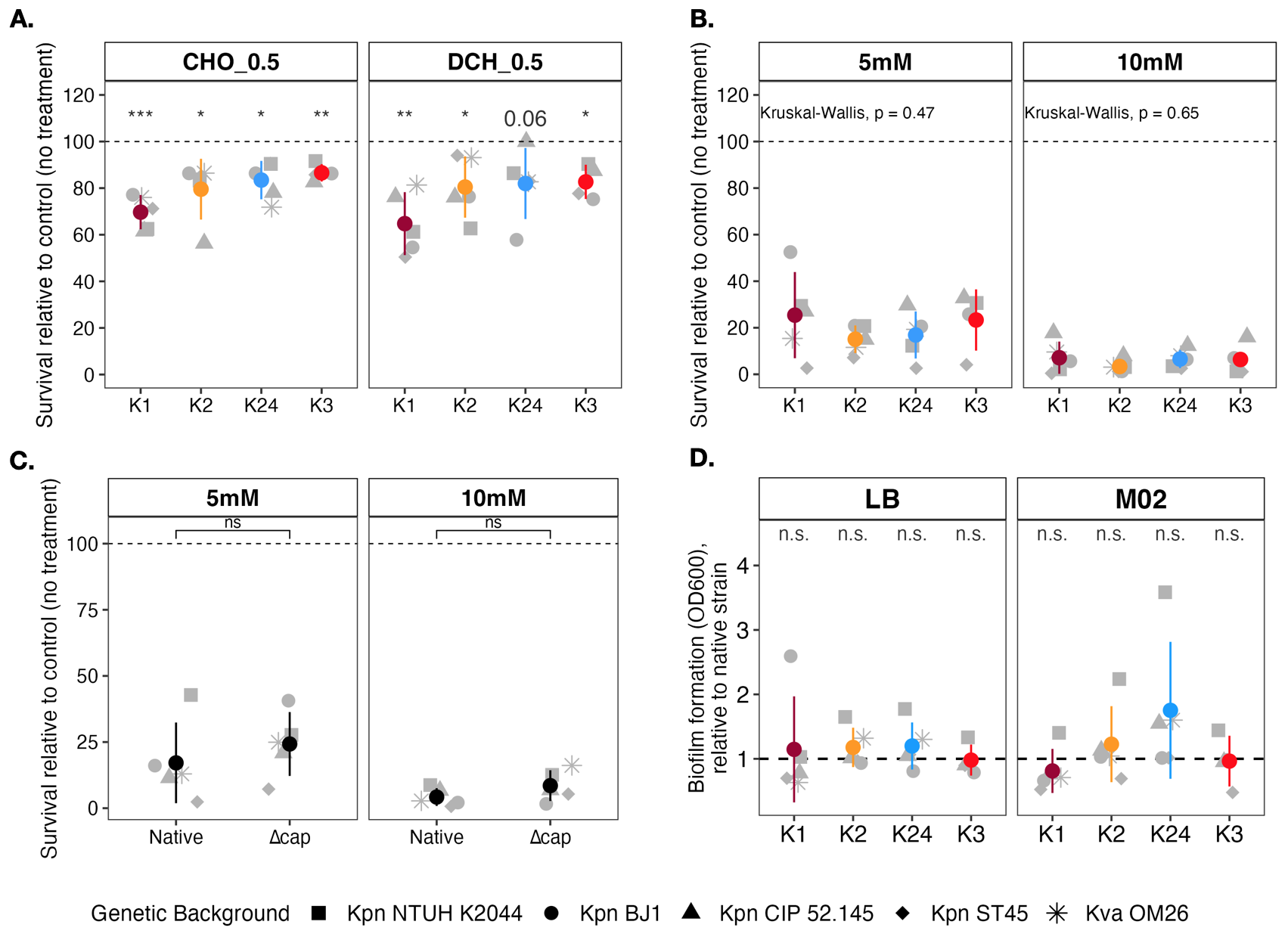


**S8 Fig. Survival to biotic stress of capsule-swapped strains. A-B.** Capsule-swapped strain’s survival to 0.5% cholate (CHO) or deoxycholate (DCO)(**A**) and to 5mM or 10mM H_2_O_2_ (**B**), relative to their respective untreated condition. *p < 0.05; **p < 0.01; ***p < 0.001, one-sample t-test, difference from 100. **C.** Native capsulated strains and their respective acapsular mutant (∆cap) survival to 5 or 10mM H_2_O_2_, relative to their respective non-treated condition. ns, non-significant, two-sample paired t-test. **D.** Biofilm formation of capsule-swapped strains in nutrient-rich (LB) or nutrient-poor (M02) media, relative to their respective native strain. Shape of dots correspond to the genetic background; the K type is indicated on the x-axis and identified by the color. Each point represents the mean of at least three independent biological replicates. ns:non-significant; one-sample t-test, difference from 1. The data underlying this Figure can be found in S2 Data.
