# Supplementary material for: Plug-and-play evolution of the Klebsiella pneumoniae capsule locus enables serotype exchange across genetic backgrounds
Source: PLoS Biol. 2026 Mar 25;24(3):e3003724. doi: 10.1371/journal.pbio.3003724 (PMC13043062; doi:10.1371/journal.pbio.3003724)
Supplement: S9 Fig — A. Raw values of the hypermucovsiscosity index (HMV) of capsule-swapped strains, as measured after growth in nutrient-poor medium. B. Hypermucovsiscosity index of capsule-swapped strains relative to their respective native strain in nutrient-poor medium. The serotype is indicated on the x-axis and by the color. Gray points represent independent biological replicates and color points represent the mean of these biological replicates. Asterisk beside K loci (*) indicates the native serotype of each strain. ns: nonsignificant; one-sample t test, difference from 1. The data underlying this Figure can be found in S2 Data. (DOCX) [file pbio.3003724.s009.docx]

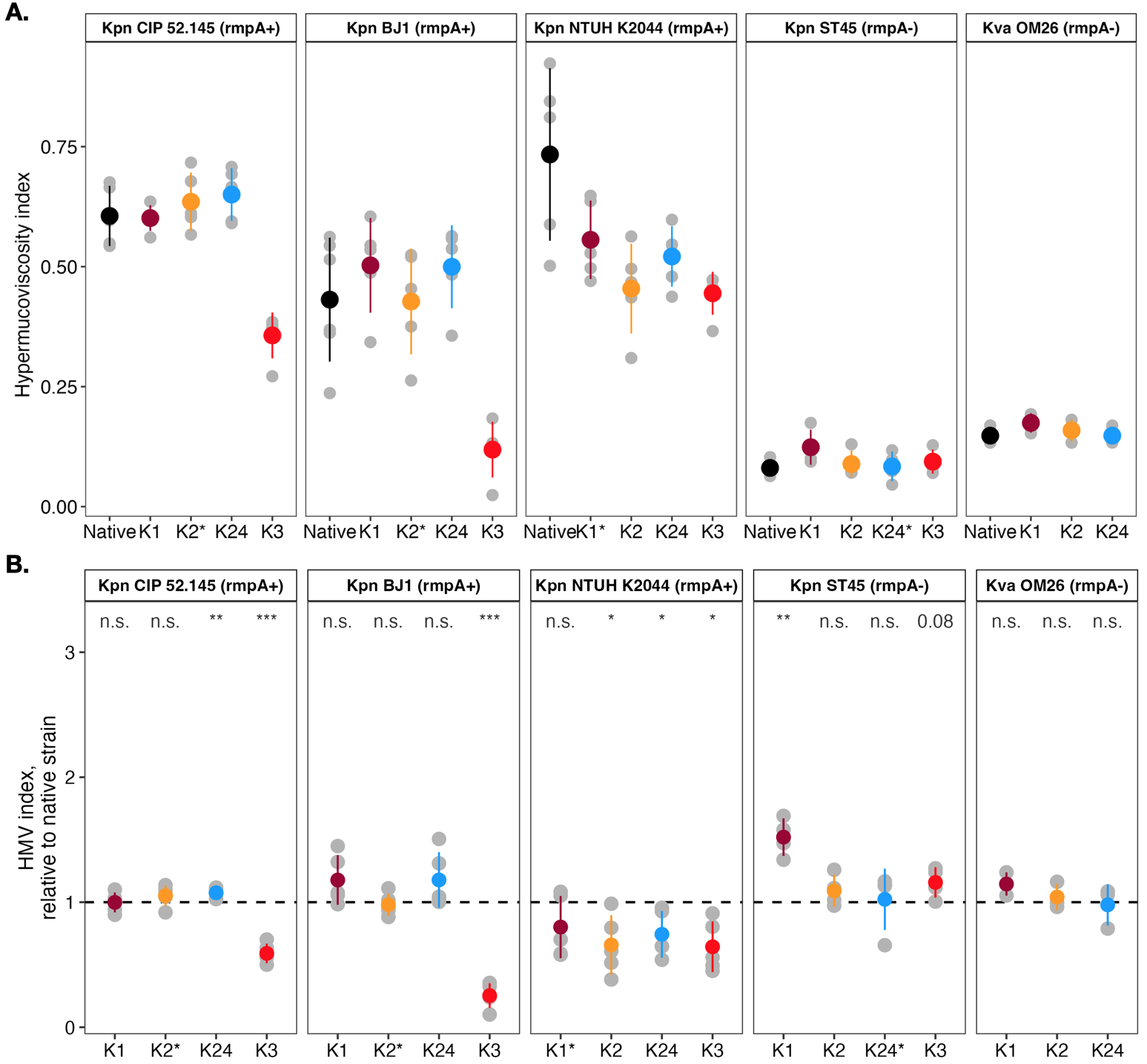


**S9 Fig. Hypermucoviscosity index of capsule-swapped strains. A.** Raw values of the hypermucovsiscosity index (HMV) of capsule-swapped strains, as measured after growth in nutrient-poor medium. **B.** Hypermucovsiscosity index of capsule-swapped strains relative to their respective native strain in nutrient-poor medium. The serotype is indicated on the x-axis and by the color. Grey points represent independent biological replicates and color points represent the mean of these biological replicates. Asterisk beside K loci (*) indicates the native serotype of each strain. ns:non-significant; one-sample t-test, difference from 1. The data underlying this Figure can be found in S2 Data.
