# Supplementary material for: Plug-and-play evolution of the Klebsiella pneumoniae capsule locus enables serotype exchange across genetic backgrounds
Source: PLoS Biol. 2026 Mar 25;24(3):e3003724. doi: 10.1371/journal.pbio.3003724 (PMC13043062; doi:10.1371/journal.pbio.3003724)
Supplement: S10 Fig — A. Capsule production measured by the uronic acid method and normalized by the OD600. B. Biofilm formation, quantified by crystal violet, after 24 hours growth in either nutrient-rich (LB) or nutrient-poor (M02) media. C. Hypermucoviscosity index upon slow centrifugation. The data underlying this Figure can be found in S2 Data. (DOCX) [file pbio.3003724.s010.docx]

**
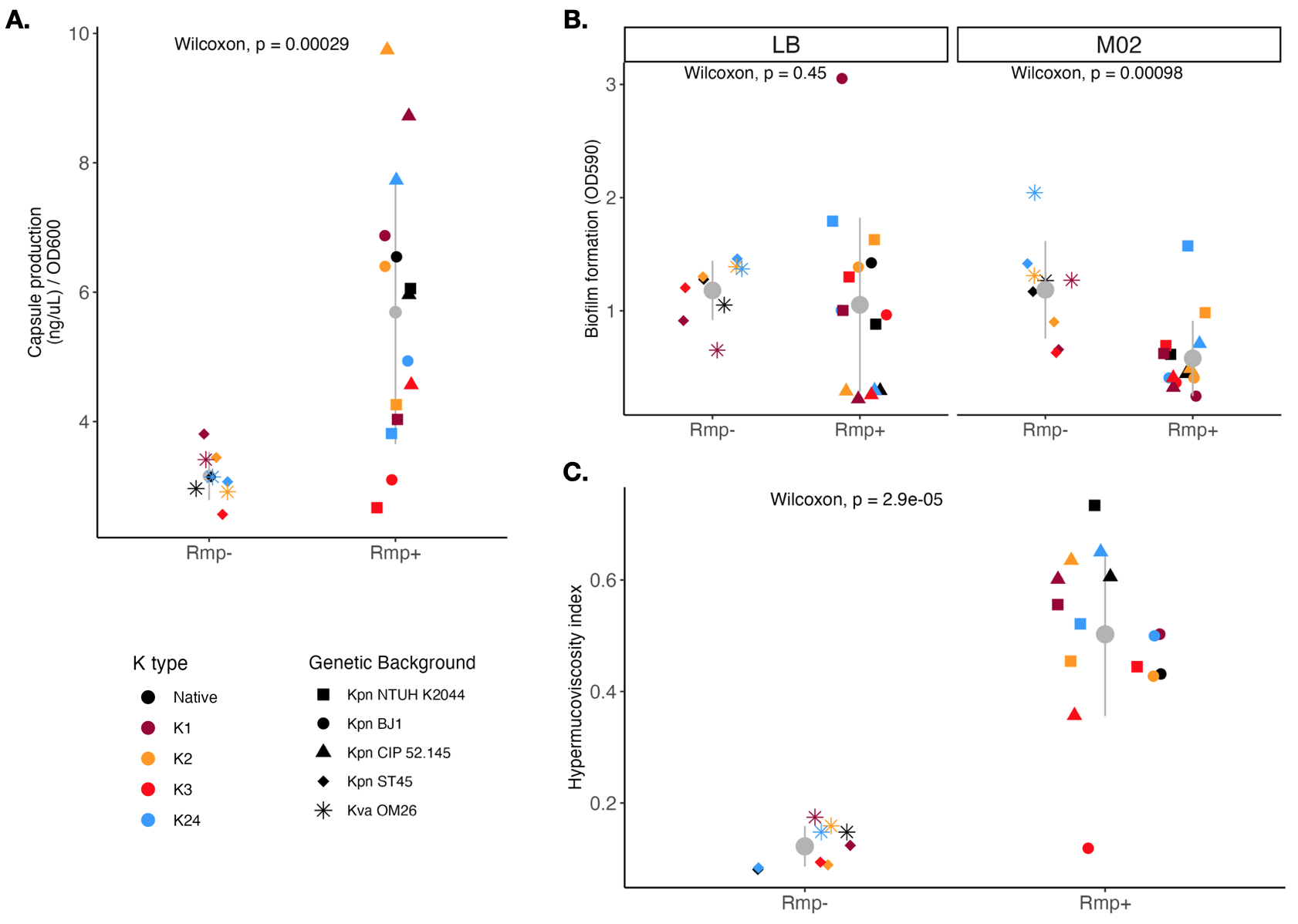
**

**S10 Fig. Experiments analyzed considering the presence (*rmp*+) or absence (*rmp*-) of the *rmp* locus in the genetic background of each strain. A.** Capsule production measured by the uronic acid method and normalized by the OD_600_. **B.** Biofilm formation, quantified by crystal violet, after 24 hours growth in either nutrient-rich (LB) or nutrient-poor (M02) media. **C.** Hypermucoviscosity index upon slow centrifugation. The data underlying this Figure can be found in S2 Data.
