# Supplementary material for: Plug-and-play evolution of the Klebsiella pneumoniae capsule locus enables serotype exchange across genetic backgrounds
Source: PLoS Biol. 2026 Mar 25;24(3):e3003724. doi: 10.1371/journal.pbio.3003724 (PMC13043062; doi:10.1371/journal.pbio.3003724)
Supplement: Attachment — Submitted filename: Rebuttal.docx [file pbio.3003724.s024.docx]

2^nd^ February 2026

Dear Dr Vázquez Hernández,

We thank you for the opportunity to revise our manuscript and for forwarding the reviewers’ comments. We are grateful for the reviewers’ positive assessment of our work and for their constructive suggestions.

Enclosed, please find our revised manuscript, presenting new experiments, along with a detailed rebuttal letter addressing each comment point by point.

Following comments of both reviewers #1 and #3, we present measurements of capsule thickness by phase contrast microscopy. These positively correlate with our capsule measurements done by the traditional uronic acid method. We also performed string tests for hypermucoviscosity as requested by reviewer #1. We have also added the sequencing analyses of capsule-swapped strains in the supplements and performed microscope analyses of evolved clones with irregular cell segmentation, as suggested by reviewer #2. Finally, we have thoroughly addressed the concerns of reviewer #3 regarding the methodology.

We believe that the revisions have strengthened the manuscript and improved its clarity and overall quality.

We appreciate the time and effort invested by you and the reviewers in the evaluation of our work, and we hope that the revised version is now suitable for publication in the journal *Plos Biology*.

Thank you for your consideration,

Julie Le Bris

**Reviewer #1:**

The work systematically investigates the functional and fitness consequences of engineered capsule swaps across diverse genetic backgrounds, combining transcriptomics, short-term evolution, and phenotypic assays. The main finding is that capsule loci can function as modular, "plug-and-play" units with minimal disruption to the host, which provides a plausible explanation for their widespread exchangeability in nature. The study further reports that key virulence traits associated with specific serotypes are conserved after transfer, and that adaptation to capsule cost follows general characteristics rather than serotype specificity. The manuscript is logically organized, and its findings provide important insights into the evolutionary consequences of capsule exchange. However, all evidence is derived from in vitro experiments. The consequences of capsule swapping in an in vivo host environment remain unaddressed. There are several issues in the manuscript.

We thank the reviewer for the thorough and detailed review of our manuscript. We appreciate the time and effort invested in reviewing our work.

We acknowledge that our results are derived from in *vitro* experiments. Our motivation for this work was to understand the exchangeability of bacterial capsules from an eco-evolutionary standpoint, which we have addressed thoroughly. The consequences of capsule swapping in virulence has already been addressed (with most of the serotypes included in this study) using different models in a previous publication referenced in the text (<https://doi.org/10.1371/journal.ppat.1010693>). They show heritability of virulence with capsule serotype which reinforces the “plug-and-play” mechanism evidenced here. We thus believe that repeating those experiments would not add significant novelty. We also consider that they are out of the scope of this study, which is focused on bacterial evolution and not specifically on infection by Kpn.

Main points:

1. Direct evidence (e.g., by electron microscopy or capsule staining) is needed to support the claim that higher capsule production in K1/K2 corresponds to greater physical thickness, as the manuscript consistently equates production levels with thickness without morphological or quantitative validation. Additionally, the discussion should address the molecular basis for serotype-dependent production.

We agree with the reviewer that thickness was not measured directly.

We now provide new experiments using phase contrast microscopy and India ink staining to measure the capsule. Briefly, we imaged all capsule swapped strains of three different genetic backgrounds (NTUH K2044, CIP 52.145 and ST45) and processed the images using ImageJ. We used the exclusion area around each cell as a measure of capsule thickness. Of note, we have previously shown that cell size *per se* (as measured by flow cytometry) does not vary in capsulated cells when compared to non-capsulated-cells (Figure 1D PMID **40905702**). Thus, changes reported here of the exclusion area are due exclusively to capsule thickness.

We confirm that uronic acid measurements almost perfectly correlate with the new measurements of capsule thickness. We realized that the image analyses method also presented some limitations, as capsule thickness could not be confidently evaluated in strains with very little capsule production (see Figure S3). These were thus excluded from capsule thickness analyses. However, the new measurements of capsule thickness also correlate with another method which uses cell volume as a proxy for capsule thickness. This method was first used in a previous manuscript from the lab, using capsule-swapped strains (Figure S1 of PMID: 38448399). In conclusion, uronic acid, cell volume and image analyses all positively correlate and establish a capsule production hierarchy: K1> K2> K24 >K3

These new analyses are now shown in Figure S3, and the methods are described in the appropriate section (L.648-658).

Molecular basis of serotype-dependent production is now discussed (L.452-459).

2. Are Figure 1B and 1C generated from the same dataset and processed differently? If so, why does Figure 1B show the highest K2 capsule production in the Kpn CIP 52.145 background, while Figure 1C indicates the highest production for K1 in the same background?

Figures 1B and 1C were indeed generated from the same dataset, and the underlying raw data are identical in both panels.

In the specific case of the Kpn CIP 52.145 background, capsule production levels are qualitatively, but not significantly higher in K2 than in K1 as shown in Figure S2. In all other strains, the hierarchy K3 < K24 < K2 < K1 is respected and was thus chosen as the most consensual trend. Detailed visualizations of these serotype- and background-specific capsule quantifications are provided in Figures S2B–C, and in the new Figure S3. This has now been clarified in the legend of Figure 1 (L.162-163).

3. The results in Figure 2 indicate that the capsule gene expression is fairly constant independently of the genomic context. However, findings in Figure 1 show that capsule production is genetic background-dependent. This appears somewhat contradictory. How can the stability of capsule gene expression across different backgrounds be reconciled with the significant differences in capsule production?

We thank the reviewer for raising this point and agree that clarification is needed. However, Figure 2 does not indicate that capsule gene expression is constant across different genetic backgrounds, but rather constant within a given strain (same genetic background). Due to statistical constraints inherent to the RNA-seq analysis, comparisons of capsule gene expression were restricted within a given genetic background and were not performed across different genetic backgrounds.

This has been clarified in the legend of Figure 2 and in the methods section.

We also discuss serotype-dependent production of the capsule (see comment #1) in L.450-457.

4. Regarding Figure S6B, the native Kpn BJ1 strain is K2 serotype. Similarly, there is a capsule-swapped strain in the Kpn BJ1 background that also carries the K2 locus. However, in the short-term evolution experiment, the swapped K2 strain showed a significant increase in acapsular clones. What might explain this observation?

We thank the reviewer for this observation. We also noted that the swapped K2 strain in the BJ1 background showed a higher frequency of acapsular clones. Statistical tests (Wilcoxon signed-rank test) comparing the area under the curve between parental Kpn BJ1 and Kpn BJ1 K2 capsule-swapped strain showed marginal but significant differences, p=0.048. This was not significant for all the other pairs (see Figure below). This is most likely due to randomness inherent to evolutionary processes, and to the limited number of populations in the evolution experiment.

Legend: Area under the curve (AUC) corresponding to the depletion of capsulated clones throughout a 15-day evolution experiment.

5. In the investigation of the hypermucoviscosity phenotype conferred by different capsule types across genetic backgrounds with or without the rmp locus, was the entire rmp locus systematically analyzed? Figure S8 only presents data for rmpA, yet according to literature, the rmpD gene is also crucial for the mucoid phenotype. Additionally, the string test should be performed to provide direct phenotypic confirmation of hypermucoviscosity.

We thank the reviewer for this suggestion. We have included additional data in Supplementary data S3 (generated using the -m klebsiella__rmst module of Kleborate v3). This table now contains information on the complete *rmp* locus, including lineage, sequence type, and allele numbers for *rmpA, rmpD, rmpC*.

As suggested by the reviewer we have also performed a string test, we provide the results in supplemental material, but we prefer not to analyze these further This provides only a qualitative assessment rather than a quantitative and is quite sensitive to environmental variations. Indeed, there are references claiming that “*This [sedimentation assay] objective assay is more quantitative and reproducible than the string test.”* (Mike et al. 2021) and *“Centrifugation was chosen over the string test because it is semiquantitative, whereas the string test is purely qualitative.*” (Walker et al. 2020). Also, the qualitative nature of string tests precludes statistical analysis, limiting our ability to detect subtle changes.

This is now included in the methods (L665-668) and supplemental material (Supplementary data S6.).

6. Only the K1 capsule confers a conserved hypermucoviscous phenotype across genetic backgrounds does not support the broad conclusion that "hypermucoviscosity was conserved across genetic backgrounds." Therefore, thise statement appears overstated and lacks the precision.

We apologize for the confusion. What we meant is that K1 confers a HMV+ phenotype, but the opposite is also true. Some serotypes such as K3, in originally HMV+ genetic backgrounds with *rmp*+, systematically lead to HMV- strains, measured both by the sedimentation assay and the string test. We thus meant that the sign of HMV (whether + or -) was conserved across the genetic background.

We have rephrased the text to avoid confusion.

7. The conclusions are based solely on in vitro evidence, without an assessment of the consequences of capsule swapping in an in vivo host environment.

Our work aims to uncover the broader evolutionary consequences on bacterial fitness and physiology of capsule exchangeability, which remain largely unexplored, whilst providing an explanation for their pervasiveness. Previous studies have already focused on virulence inheritance. More specifically, Huang et al. (PMID: 35914009) used similar approach to test the impact of capsule swapping on virulence and clearly show that some capsule locus alone can encode specific virulence functionalities, providing *in vivo* proof to the plug-and-play mechanism of capsule evolution.

This is discussed in L.489-495.

Minor points:

1. There is no citation for Figure 4B in the manuscript.

Sorry for the oversight, this has now been corrected.

2. The presentation of some data lacks the optimal clarity. For instance, representing bacterial growth curves solely by the area under the curve (AUC) metric, rather than showing the raw OD values over time, obscures the actual growth dynamics.

We agree, and the raw absorbance values over time are now presented in Figure S6A.

------------------------------------

Reviewer #2 (Alvaro San Millan):

In this study, Le Bris and colleagues analyze the phenotypic and transcriptomic effects associated with capsule swapping in different strains of Klebsiella pneumoniae. The capsule locus is commonly exchanged between strains in this species, and the authors hypothesized that capsules may behave as modular, "plug-and-play" units, that produce minimal alterations in the new bacterial hosts while maintaining their functions. In order to test this hypothesis, they constructed a collection of five wild-type trains carrying four different capsule types each, and performed transcriptomic, fitness and evolution assays. Their results convincingly showed that the capsule locus produces minimal effects on the transcriptomic profiles and fitness of the hosts, but conserved serotype-specific traits, such as biofilm formation and hypermucoviscosity. In general, I think this is a timely and relevant study. I particularly appreciate the technical difficulty of constructing the strains, as well as the elegant and robust experiments used. I enjoyed reviewing the paper, and I think it will be of interest for the general readership of PLoS Biology. I provide a few specific comments below:

We thank the reviewer for the positive evaluation of our manuscript. We are grateful for the constructive comments and for the reviewer’s appreciation of the significance of our results.

-Construction of the strain collection. The authors sequenced the genomes of the final strains to ensure that no off-target mutations were acquired during the process, however, they did not present the results. I guess some mutations may have accumulated, and it may be interesting to have a table with those.

We now provide the results of the sequencing analysis for the capsule-swapped strains (including the intermediate acapsulated mutant) in Supplementary data S5.

-In general, the results with serotype K1 tend to be more variable across different experiments. Could this be due to a rapid rise in capsule-less mutants in rich medium during the experiments?

Thank you for this comment, we do notice this. We believe that increased variability of K1 strains across experiments could be explained both by the inherent difficulty to manipulate strains with a hypermucoid phenotype characteristic of K1 strains, but also by the strong positive selection of capsule-less mutants in rich medium (as suggested by the reviewer).

-Lines 352-354, the authors could easily test the presence of long cells by flow cytometry (using forward scatter values as a proxy) or microscopy.

We thank the reviewer for this suggestion. We performed phase contrast microscopy of the ancestral strain and of selected evolved clones carrying the *tatC* mutation (see Figure below). We confirm that populations with these mutations can appear longer, but this is due to the fact that TatC defective mutants display altered or no translocation between the cytoplasm and periplasm of proteins AmiA and AmiC. These are two amidases required for cell separation upon cell division.

Legend: Phase contrast microscopy of ancestral strain Kpn ST45 and one randomly chosen clone from evolved population #1 with a mutation in *tatC*.

We do not wish to further elaborate on this specific result in this manuscript as segmentation defects in *Enterobacteriales* as a result of mutations in the Tat translocation system are already fairly well described (see PMID: 21298091, PMID: 15347649). Also, our team has already reported these segmentation defects in more detail in a recent study (Figure 4C -  <https://doi.org/10.64898/2025.12.17.694638>) from another evolution experiment (different settings). Consequences of such segmentation defects will be pursued further as part of other projects, and we believe are not specific to the swaps but adaptation to the growth media used in the experiment.

-lines 388-391, this sentence is confusing.

We rephrased this sentence (L.402-403).

-Figure 5, any idea why Kpn BJ1 is producing so much more biofilm with the K1 capsule?

This also appealed to us as an interesting result for us, but at this time we cannot propose a strong hypothesis, other than specific physico-chemical properties between the K1 capsule and other exopolysaccharides and/or matrix components secreted by the BJ1 strain.

This is now mentioned in lines 394-396.

-Another interesing example of "plug and play" of a macromolecular system with no phenotypic and transcriptomic effects that could be commented in the discussion can be found here https://urldefense.com/v3/__https://academic.oup.com/nar/article/52/20/12565/7816861__;!!JFdNOqOXpB6UZW0!uGsHDjquhINDnZI9tCyDLRCsIpdBjiba9-Emjy1Ud5V-VKK6oul37zpKXXBc7EYqLUIaCbrA9BucouIoVA$

Thank you for this suggestion which had slipped our attention. This manuscript is now referenced in the text (L.68-70).

Alvaro San Millan

------------------------------------

Reviewer #3:

This manuscript by Le Bris, et al. (PBIOLOGY-D-25-03672R1) presents a series of experiments in which they took several Klebsiella strains and introduced 4 different capsule loci into each background to evaluate fitness and other phenotypic consequences of the swapping. An initial observation was that some loci yielded more capsule irrespective of strain background, and that some strains produce more capsule irrespective of capsule type. The authors then examined transcriptomics, growth curves, in vitro competition, rate of capsule loss due to environmental pressures, and phenotypes associated with virulence. The collective observations indicate that swapping capsule loci has very little if any impact on the tested phenotypes. These results suggest that the capsule loci are not broadly restricted to certain strain backgrounds, and "easily integrated" (my phrase) into most strain strains without causing major shifts or the need for adaptation. Previous studies have examined capsule swapped strains but have focused on the role of capsule type in virulence. This manuscript presents a novel angle, trying to understand the evolution of capsule and how horizontal transfer of the capsule locus impacts the recipient strain. This work advances the field of both capsule and Klebsiella biology, suggesting that the number of strain background/capsule type pairs among Klebsiella strains is a consequence of the lack fitness costs associated with interchanging capsule loci. A major strength of this study is in the breadth of strain backgrounds and capsule types examined. Although many of these results feel "negative", the findings are collectively fascinating and important. This manuscript is well-written and well-organized with sound conclusions based on the data presented. A few comments that I hope the authors will find helpful are listed below.

We thank the reviewer for their positive assessment of our work and for recognizing its contribution to the field. We appreciate the encouraging feedback and are pleased that the reviewer found the manuscript clear and valuable.

Major Comments

1. Throughout the manuscript, please make axis labels on graphs larger. They are proportionally very small relative to the graph/image size, and in many cases nearly unreadable.

We acknowledge some labels were small. Accordingly, we have modified our figures. We hope this is now satisfactory.

2. One finding I found particularly intriguing is that strain 52.145 with K1 had so many differentially expressed genes, but none of the others had any effect even close to this. I was hoping for more detail/speculation in the discussion, but this was just glossed over. I was not surprised to see that most swaps did not result in much change in expression as the capsule loci do not contain transcriptional regulators, so this result really stood out to me.

We thank the reviewer for this comment and agree that this was somewhat an unexpected result. We now provide a little more detail and further clarify this in the text (L. 239-240).

Also, if I understand the differences between Figs 2B and 2C correctly, it suggests there are~300 CPS-related genes that are differentially expressed? (a) if this interpretation is wrong, then I think the text may need some clarification, (b) if correct, how are CPS-related genes determined? This seems very high to me. Perhaps a bit more detail on which genes were most highly DE would be of interest to other readers!

We apologize for the lack of clarity The reviewer is correct, ~300 CPS-related genes would indeed be very high. Figure 2B reflects the total number of genes differentially expressed (CPS-related and non CPS-related) with results expressed in Fold Change, while Figure 2C reflect the number of non CPS-related genes differentially expressed this time expressed in |log_2_FC| (corresponding to Fold Change >=4). We have reformulated this in the figure legend and hope it is now clearer.

Some details on most differentially regulated genes (KEGG pathways) are presented in L235-239. Also, a new supplemental table with all significantly regulated genes is presented (Supplementary data S1).

3. Fig. 2, why was a K1 strain with swapped CPS not included in the differential gene expression analysis? It struck me as odd to include two K2 strains and omit the K1.

We thank the reviewer for this question. The NTUH strain and its respective swapped strains were not included in the differential gene expression analysis due to practical constraints on the number of RNA samples that could be sequenced. We prioritized including the two K2 strains because they are genetically distant on the *Klebsiella* phylogenetic tree and, based on previous work in our lab, could be expected to display more divergent transcriptional responses. Including both K2 backgrounds allowed us to capture the potential range of variation in capsule gene expression across distinct genomic contexts.

As a complement to our response, during our preliminary experiments, to optimize the RNA-sequencing protocol, we did send a K1 swapped with K2 (and complemented K1). Our results did not differ from those observed with other genetic backgrounds, that is, we did not observe any large changes in expression. The data are not presented here as the full set of swapped strains for the K1 strain NTUH-K2044 is not available.

4. The paragraph/section beginning on line 305 (and lines 301-303) describes capsule thickness, but thickness was never measured—only amount of capsule. I would encourage limitation of linking amount to thickness.

This concern was also raised by reviewer #1. Please, see our answer to reviewer #1, comment #1.

5. Regarding the biofilm data in Fig 5, I would like to know what the relative biofilm production looks like between the strains (particularly between HMV+ and HMV- strains), not just ratios of the impact of the varying K types. Could the authors provide this in a supplemental figure? A simple bar graph of OD595 would suffice.

We now provide a graph separating the strains according to their *rmp* status (presence/absence).

We do not observe any influence of *rmp* presence in rich media, but we do observe a decrease of biofilm formation when grown in poor-media in *rmp*+ strains. This is now included in Figure S10B and mentioned in the results (L.413-423).

Legend: Biofilm formation during growth in either nutrient rich (LB) or poor (M02) media. Biofilm formation was quantified with crystal violet. Results are presented separated by their *rmp* genotype.

6. The sedimentation resistance values for ST45 and OM26 (Fig. S8) are surprisingly high given they are rmp negative. I'm concerned this may have to do with the specific protocol applied (see concern below regarding this), and therefore am somewhat concerned about the interpretation of the data. HMV negative strains should have a post-spin OD600 value below 0.15-0.2. Have the authors verified these strains do not contain wzc mutations that might lead to elevated resistance? Was a capsule mutant included in the assays? This strain should have a post-spin OD600 of nearly zero, so if this strain is much above 0.1, I would revisit the protocol parameters.

These strains do not have a known mutation in *wzc*.

Concerning differences in absolute values, we would like to emphasize that this experiment was performed in M63B1 supplemented with 0.2% glucose (M02) to avoid accumulation of non-capsulated mutants.

We however thank the reviewer for this observation as it allowed us to realize that we had an issue with the centrifuge settings (rcf) and we apologize for our oversight. During the time of these experiments, we changed labs (and centrifuges). This resulted that the settings (rcf) were not the same between the first and the second set of tested swapped strains. This explains the differences in the raw values. In light of this comment and another below (comment 10), we repeated the experiments for the full set of strains that seemed to have unusually low or high values (Kpn BJ1; Kpn ST45; Kva OM26). All figures have been modified and all stats recalculated to take into account only values performed at the same (and adequate) rcfs. Despite the settings altering the raw values of these strains, these did not qualitatively affect the observed trends.

7. Although I generally thought this manuscript was well-written, there are numerous typos and a few issues with grammar that I found led to some confusion on my part. I am already late submitting this review, so I'm not detailing them, but I do think it might be helpful to have a fresh set of eyes give it a read through!

Thank you for the heads up, we appreciate it. We have thoroughly verified our text and we hope that all issues have been resolved.

I have several concerns about information in the Materials & Methods:

8. I noted a statement that raised two concerns. Lines 594-595 states that a culture was mixed with zwittergent, then the supernatant was discarded to quantify only CPS attached to the cell. (1) However, I believe the zwittergent removes attached CPS. If this information is true of the steps performed, that the bulk of the CPS was discarded, and I'm not confident that what is reported is close to an accurate measure of true CPS production. The units reported differ from 2 lab-to-lab, so I cannot evaluate if these are in line with other publications.

We apologize for this text mistake, indeed the supernatant was kept and washed with ethanol to extract polysaccharides.

This has now been corrected.

(2) As there is a significant amount of CPS that is released into the supernatant, I'm not sure that the stated goal of measuring only cell-attached CPS is the most accurate representation of CPS production. While I do not think these particular data will alter the other findings reported here, I do feel this needs to be addressed. The authors should clarify their protocol steps and justify why measuring cell-associated CPS was chosen over total CPS.

We agree with the reviewer and our choice is now justified in the methods section (L629-630).

9. A separate concern about the CPS quantification is the apparent calculations. Line 602, it is stated that OD520 was divided by OD600. The normalization step should be performed after OD520 is used to calculate CPS concentration. If the authors have done the math both ways and show that it does not matter, I would be satisfied, but might suggest they rephrase more simply that CPS production was normalized to culture OD to avoid raising concerns from other readers. I place this in the "major" concerns section as it needs to be resolved/clarified for accurate reporting, but I can see that it likely will not change interpretation of the data.

We thank the reviewer for this suggestion. We have now rephrased this sentence and mention that to normalize for number of cells, the capsule production was normalized to culture OD600. (L643-644).

10. I have another procedural question regarding the HMV assay. Line 616 states the cultures were spun at 2500 rpm. This is not an ideal unit as rpm-to-centripetal force varies between centrifuges. Please report this as "# x g" to allow comparison with other studies. Also, please clarify "background noise was subtracted" (line 618), and if the cultures were normalized to a constant OD prior to centrifugation.

We clarified this part of the methods.

As mentioned above, and now clarified in the methods, we report the #g and specify that we normalized capsule production to OD600nm to account for cell density variations.

Minor Comments

1. Lines 83 & 90, I understand the need for simplification, but I am concerned that use of the term "serotypes" to describe the different capsule types since not all have actually been serotyped and assigned a K type designation based on sequencing alone. If this is only to refer to the K types in this study, it would be acceptable but this should be stated a bit more clearly.

We thank the reviewer for this comment. To address this concern, all instances of “serotype” have been replaced with “K type” throughout the manuscript, and the title was modified accordingly. We have clarified in the text that “K type” refers to the capsule types analyzed in this study, which were indeed assigned based on sequence information rather than classical serological typing (l.86-88).

2. Fig. 1A needs definitions/symbols to identify the sugar precursors & color scheme in the heat map. In figs. 1B&C, it seems odd to me to present these data as a line graph because each has different variable on the X-axis, rather that a single variable (e.g. time, concentration). I wonder if stacked bar graphs wouldn't be more appropriate for conveying the key points?

Modifications to clarify Figure 1A were done as suggested. Concerning Figures 1B and C, we feel that the general trends are most important and should remain as a main figure, and all the individual data for each genetic background – serotype combination are detailed in the supplementary Figure S2.

3. Fig S4, the scale is very important in this figure as it varies in each plot and needs to be easily visualized

We agree that the use of different scales can be misleading. However, axis coordinates are not directly comparable between samples because they are generated from distinct analyses. Specifically: i) for PCA plots within the same panel, each corresponds to an independent analysis performed for each genetic background; ii) for PCA plots across different panels of the same set of strains, each PCA is based on a different gene set (A. core-capsule, B. core capsule without ∆cap strain, C. core-genome).

To improve readability, we have increased the font size of labels and scales.

4. Fig S5, 52.145-K3 has an unusual growth pattern—is this reproducible?

We agree, and we confirm it is reproducible. In Figure S5A, we did not show the error bars for clarity purposes but please find here below the data with the appropriate standard deviation (n=5).

Legend: Growth curve of Kpn CIP 52.145 WT and K3 swapped strain. Growth was measured both in nutrient rich (LB) and nutrient poor (M02) medium. Error bars indicate standard deviation across five independent biological replicates, each calculated as the mean of three technical replicates.

5. Lines 310-311, the logic for performing the in vitro evolution experiment feels countered by the absence of a fitness cost from the growth curves. I do think this is an important experiment with interesting findings, but I would suggest setting up the justification a bit differently to avoid others being confused (as I was).

We thank the reviewer for the suggestion and modified the text to make it clearer (L316-318).

6. Lines 349-350, Mike, et al 2020 (reference #38) would be appropriate to include here.

Sorry for the oversight, the reference was included (L.359)

7. Several times, the phrase "precultures were grown overday". I do not understand what this means and would appreciate some clarity. Were colonies inoculated in the morning, then subcultured late in the day? Were these subcultures of overnight cultures?

Exactly, colonies were inoculated in the morning in LB and subcultured overnight in minimal media (M02).

This is clarified in the text.

8. The method for bile salt survival seems a bit unusual—is there a reference for this approach? More typically, there is an incubation with the target agent for a period of time, then dilution and plating rather than plating on solid medium with the agent.

We thank the reviewer for this comment. This method was previously used and results published by our lab and others working on other bacterial species (PMID: 33653142, PMID: 38330058, PMID: 40874615). This approach allows for direct evaluation of growth capacity in the presence of bile salts, rather than short-term survival following exposure.

This is now appropriately referenced in the methods section.

9. In the section describing introduction of the mCherry gene (beginning line 652), as written, some terminology is incorrect. It reads to me that this is an insertion event via homologous recombination within the glmS gene, rather than specific integration at the Tn7 site. Please clarify by stating more precisely where the gene was integrated.

We apologize for this. We have now clarified this part of the methods (L.711-715).

10. The statement beginning on line 388 that K1 increased HMV is consistent with the observations in Salisbury, et al. (PMID: 41363447), however the contrasting point that K3 reduced HMV is very intriguing. Can the authors speculate on why this may be? Are there notable differences that might contribute to this?

We thank the reviewer for pointing this out. We have no experimentally validated answer but we hypothesize it could be due to specificities of our K3 locus, notably truncation of the *cpsACP* gene or a particular sequence in key capsule genes like *wzc* that could decrease the HMV. This contrast with the results observed in (PMID: **39702763**), where K3 strains have overall high HMV.

This is now mentioned in the text (L.407-411)

I think some additional attention to differences observed between rmp+ and rmp- host strains might also be informative.

We now provide some additional graphs comparing the rmp+ and rmp- strains (regardless of the HMV phenotype) in Figure S10.

11. Line 352, Walker, et al. 2020 (PMID: 32963003) would be a more appropriate reference or additional reference to include here.

We added the reference (L.398).
